# SlHSP17.7 Ameliorates Chilling Stress-Induced Damage by Regulating Phosphatidylglycerol Metabolism and Calcium Signal in Tomato Plants

**DOI:** 10.3390/plants11141865

**Published:** 2022-07-18

**Authors:** Yuanyuan Wu, Shuwen Lv, Yaran Zhao, Chenliang Chang, Wei Hong, Jing Jiang

**Affiliations:** 1 College of Horticulture, Shenyang Agricultural University, Shenyang 110866, China; 20182000029@stu.syau.edu.cn (Y.W.); 2020220375@stu.syau.edu.cn (Y.Z.); 2020220380@stu.syau.edu.cn (C.C.); 2 Institute of Vegetable Science, Liaoning Academy of Agricultural Sciences, Shenyang 110161, China; shuwenlu@163.com; 3 Shenyang Institute of Technology, Shenyang 113122, China; wei_9090@163.com; 4 Key Laboratory of Protected Horticulture of Education Ministry, Shenyang 110866, China

**Keywords:** tomatoes (*Solanum lycopersicum* L.), chilling stress, SlHSP17.7, phosphatidylglycerol, CBF, reactive oxygen species

## Abstract

Tomatoes (*Solanum lycopersicum* L.) are sensitive to chilling temperatures between 0 °C and 12 °C owing to their tropical origin. SlHSP17.7, a cytoplasmic heat shock protein, interacts with cation/calcium exchanger 1-like (SlCCX1-like) protein and promotes chilling tolerance in tomato fruits (Zhang, et al., Plant Sci., 2020, 298, 1–12). The overexpression of SlHSP17.7 can also promote cold tolerance in tomato plants, but its specific mechanism remains unclear. In this study, we show that the overexpression of *SlHSP17.7* in tomato plants enhances chilling tolerance with better activity of photosystem II (PSII). Metabolic analyses revealed that SlHSP17.7 improved membrane fluidity by raising the levels of polyunsaturated fatty acids. Transcriptome analyses showed that *SlHSP17.7* activated Ca^2+^ signaling and induced the expression of C-repeat binding factor (CBF) genes, which in turn inhibited the production of reactive oxygen species (ROS). The gene coexpression network analysis showed that SlHSP17.7 is coexpressed with SlMED26b. *SlMED26b* silencing significantly lowered OE-HSP17.7 plants’ chilling tolerance. Thus, SlHSP17.7 modulates tolerance to chilling via both membrane fluidity and Ca^2+^-mediated CBF pathway in tomato plants.

## 1. Introduction

Tomato (*Solanum lycopersicum* L.) is an important economic crop cultivated worldwide. However, tomato plants are susceptible to abiotic and biotic stresses, which affect both yield and quality. Among the different abiotic stresses, cold stress limits crop productivity [1,2]. Indeed, the growth and development of tomato are hindered at temperatures lower than 10 °C [3]. Under low temperatures, tomato fruit development slows down, deformities in the fruit increase, and the quality of appearance and nutritional value significantly decrease [4]. After exposure to cold stress, the photosynthetic rate in the tomato plant decreases, lipid peroxidation in the chloroplast membrane increases, chlorophyll, carotene, and lutein are degraded, and the fluidity of the cell membrane and the activity of membrane binding enzymes decrease [5]. Freezing-sensitive tomato exhibits a complete C-repeat binding factor (CBF) pathway, only LeCBF1 ((Lycopersicon esculentum CBF1) could be induced by low temperatures, and the overexpression (OE) of LeCBF1 improves low-temperature tolerance in tomato plants [6,7]. CBF1 and CBF2 are upregulated in the chilling acclimation process in tomatoes. Although CBF3 is not included in the differentially expressed genes, a qPCR analysis showed that CBF3 is also induced at sub-optimal temperatures [2]. The HY5 and MYB15 transcription factors enhance cold tolerance by activating the expressions of CBF1, CBF2, and CBF3 in tomato [8]. However, limited studies have been conducted on the regulation of low-temperature tolerance in tomato leaves and the possible impact on both the yield and quality of tomato fruit under cold stress.

Environmental stress can decrease crop yield and quality. Members of the small heat shock proteins (sHSPs) family play an important role in stress response. Plant sHSPs are encoded by nuclear multigene families and are localized in the cytoplasm, chloroplasts, nucleus, endoplasmic reticulum, peroxisome, and the mitochondria [9]. The expression levels of certain members of this family significantly increase under stress caused by high and low temperatures [10], and these proteins usually do not require adenosine triphosphate (ATP) to bind to unstable proteins and stabilize them. Furthermore, sHSPs are involved in the transmembrane transport of proteins, as well as the assembly and folding of new peptide chains. It has been shown that some sHSPs can function as molecular chaperones [11], while sHSPs genes are characteristically expressed in fruit-ripening [12]. The overexpression (OE) of *SlHSP17.7* in tomato plants enhances low-temperature tolerance [13]; yet the mechanism of cell protection by sHSPs remains nebulous.

Extensive research has been conducted to determine the role of small molecular heat shock proteins in the regulation of stress caused by high temperatures [14,15,16]. Some studies have suggested that small molecular heat shock proteins can also help mitigate the stress of low temperatures. The cytoplasmic CII small heat shock protein, PfHSP17.2, can improve the tolerance of Arabidopsis to both low and high temperatures [17]. Under low-temperature stress, transgenic plants overexpressing *sHSPs* in chloroplasts suffer less damage compared to normal plants, suggesting that sHSPs can alleviate chilling stress-induced damage [18]. Tomato plants transformed with the cytoplasmic sHSP, CaHSP18, have shown less damage to cell membranes, lower degrees of photoinhibition, and improved chilling tolerance [19]. Furthermore, a tobacco plant transfected with chloroplast sHSPs genes from pepper showed lower photosystem II (PSII) inhibition under low-temperature and dim-light conditions [20]. The expressions of *CsHSP17.7*, *CsHSP18.1*, and *CsHSP21.8* are induced by chilling stress, while *CsHSP* OE improved chilling tolerance in yeast and *Arabidopsis thaliana* [21]. However, the mechanism by which CsHSP improves the tolerance of tomato plants to low temperatures remains unclear. In the current study, we explore the signal pathways and the physiological mechanisms associated with cold tolerance using an integrative analysis of the transcriptome and metabolome. The study results could provide new strategies for breeding different varieties of tomatoes with low-temperature resistance.

## 2. Results

### 2.1. SlHSP17.7 Overexpression Improved Chilling Tolerance of Tomato Plants

**Figure 1:**
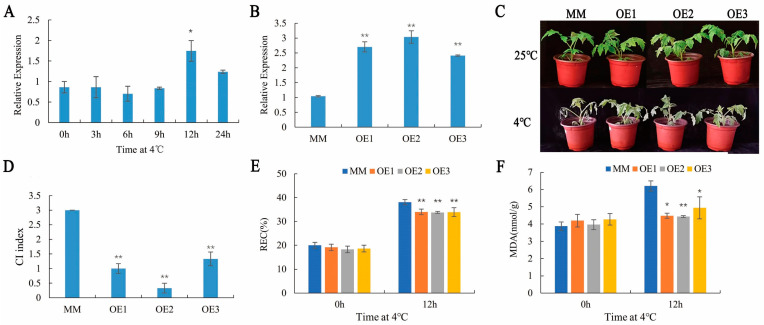
Growth of the overexpression (OE) lines and moneymaker (MM) tomato before and after chilling stress. (**A**) The expression of *SlHSP17.7* in response to chilling stress. (**B**) The expression of *SlHSP17.7* in three OE lines (t = 0 h). (**C**) Phenotype of the OE lines and MM before and after chilling stress for 7 days. (**D**) The chilling injury (CI) index for MM and the three OE lines after 7 days at 4 °C. (**E**) The REC content of the OE lines and MM before and after chilling stress for 12 h. (**F**) The MDA content of the OE lines and MM before and after chilling stress for 12 h. For (**A**,**B**,**D**,**F**), error bars represent the SD with three biological replicates (*, *p* < 0.05; **, *p* < 0.01).

We generated tomato lines with high *SlHSP17.7* expression by overexpression driven by the cauliflower mosaic virus [CaMV] 35S promoter [13]. We obtained ten independent homozygous 35S:SlHSP17.7-OE lines, from which we selected three lines (SlHSP17.7-OE1, SlHSP17.7-OE2, and SlHSP17.7-OE3) with representative phenotypes and different expression levels for further phenotypic and molecular analyses (Figure 1B). *SlHSP17.7* was significantly upregulated after a 12 h exposure to chilling stress in the MM plants (Figure 1A). So, the physiological parameters were measured after exposure to chilling stress for 12 h. The relative electric conductivity (REC) and malondialdehyde (MDA) production were lower in the OE plants than in the MM plants after exposure to chilling stress for 12 h (Figure 1E,F). However, the phenotypic changes were clear after 7 days, so the chilling injury index was calculated. After exposure to 4 °C for seven days, in the MM plants, a tendency for leaves to wilt was observed, whereas the leaves of all the OE lines remained green (Figure 1C). The chilling injury (CI) index of the OE lines was significantly lower than that of the MM plants (Figure 1D).

### 2.2. SlHSP17.7 Overexpression Improved Photosynthesis under Chilling Stress

**Figure 2:**
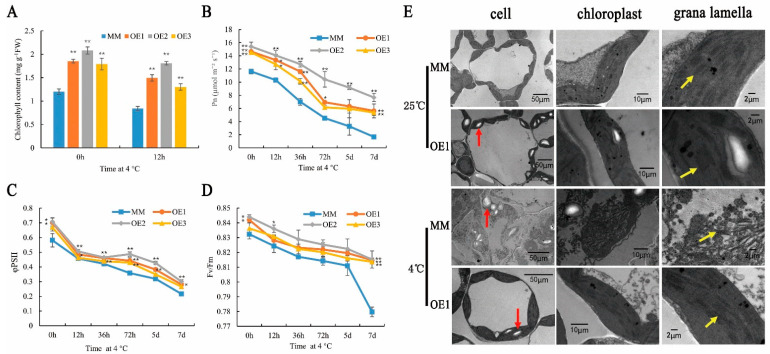
SlHSP17.7 affects chloroplast development and photosynthesis. (**A**) Comparison of the chlorophyll contents in MM and the OE lines. (**B**) Comparison of photosynthetic rate in MM and the OE lines before and after chilling stress. (**C**) Effect of chilling stress on φPSII of MM and the OE lines. (**D**) Effect of chilling stress on Fv/Fm of MM and the OE lines. For (**A**–**D**), data are presented as mean ±SD of three biological replicates (*, *p* < 0.05; **, *p* < 0.01). (**E**) Observation of chloroplast ultrastructure. The red arrows indicate the starch grains. The yellow arrows indicate the grana lamellas. Bars, 50 μm, 10 μm or 2 μm.

Under normal conditions, the total chlorophyll content and the net photosynthetic rate (Pn) in OE plants were significantly higher than those in the MM plants. After exposure to chilling stress, the total chlorophyll content and the Pn of both MM and OE plants significantly decreased compared to that in normal conditions. However, at 12 h, the chlorophyll content and the Pn of the OE lines were still much higher than those of the MM plants (Figure 2A,B). Furthermore, the actual photochemistry efficiency of PSII (φPSII) and the maximal photochemistry efficiency of PSII (Fv/Fm) of OE1 and OE2 were much higher than those of the MM plants before exposure to chilling stress (*p* < 0.05). Compared with the MM plants, the φPSII and Fv/Fm of all OE lines reached extremely significant levels at 72 h and 7 d, respectively (*p* < 0.01). This indicated that SlHSP17.7 decreases PSII photoinhibition caused by exposure to chilling stress (Figure 2C,D).

Chloroplast ultrastructure analysis showed that OE1 chloroplasts had thicker layers of grana and formed more starch than those of the MM plants. After exposure to 4 °C for 12 h, the chloroplast membranes and the grana layers of the MM plants were destroyed; the chloroplasts became round, and the starch grains were malformed. However, OE1 plants suffered less severe damage than the MM plants at low temperatures (Figure 2E). The results indicate that the OE lines were more resistant to low temperatures.

### 2.3. SLHSP17.7 Overexpression Initiated a Large Transcriptome Reprogramming under Chilling Stress

**Figure 3:**
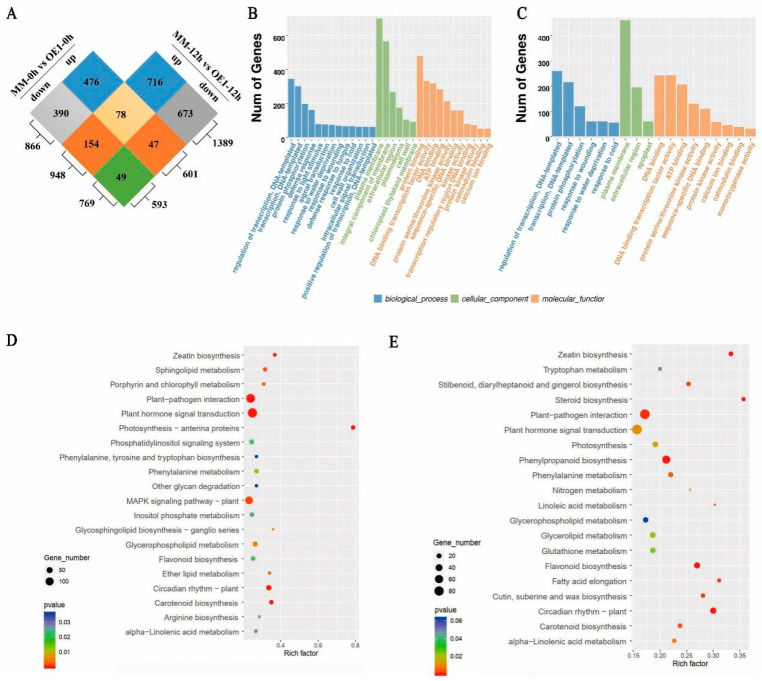
General analysis of transcriptome sequences of MM and the OE1 lines before and after chilling stress. (**A**) Venn diagram of the numbers of DEGs between MM and OE1 before (MM-0 h vs. OE1-0 h) and after chilling stress (MM-12 h vs. OE1-12 h). (**B**) The GO enrichment analysis of the DEGs between OE1 and MM before chilling stress. (**C**) The GO enrichment analysis of the DEGs between OE1 and MM after chilling stress for 12 h. (**D**) The enriched KEGG pathways of SlHSP17.7 OE1 before chilling stress. (**E**) The enriched KEGG pathways of SlHSP17.7 OE1 after chilling stress for 12 h.

To further explore the signaling pathways and the physiological mechanisms associated with the involvement of SlHSP17.7 in low temperature response, we performed a transcriptomic analysis. Subsequently, 2583 DEGs were identified between the OE1 and the MM plants according to the criteria of |log2(FC)| ≥ 1 and *p* < 0.05. Among these DEGs, 601 DEGs were upregulated and 593 were downregulated before chilling (MM-0 h vs. OE1-0 h), while 948 DEGs were upregulated and 769 were downregulated after exposure to chilling stress for 12 h (MM-12 h vs. OE1-12 h) (Figure 3A).

To validate the differential expression results, a total of 11 genes were randomly selected for quantitative real-time PCR (qRT-PCR) analyses (Table S1). These genes include heat stress transcription factor (*HSFA-1b*, *HSFA-6b*), heat shock protein (*HSP70*, *HSP90*), chlorophyll a-b binding protein (*Cab12*), catalase isozyme 1 (*CAT1*), superoxide dismutase [Cu-Zn] (*Cu/Zn SOD*), *NADPH*, *ABA 8’-hydroxylase-1*, ABA-insensitive RING protein 4-like (*RING*), cation/calcium exchanger 1-like (*SlCCX1-like*) and the mediator 26b (*MED 26b*) genes. The expression levels measured via qRT-PCR were consistent with those obtained via RNA sequencing (RNA-seq), which suggested that the RNA-seq data were reliable (Figure S1).

The results of the gene ontology (GO) enrichment analyses demonstrated that the DEGs were significantly enriched in biological processes, cellular components, and molecular functions (*p* < 0.01). In the biological processes, the top 2 GO terms were regulation of transcription and transcription, according to the number of genes before and after exposure to chilling stress. In the cellular components, the top represented GO term was the plasma membrane before and after chilling stress. Meanwhile, the chloroplast thylakoid membrane was also enriched before exposure to chilling stress. Regarding molecular function, the represented GO terms were protein binding, DNA binding transcription factor activity, DNA binding, transcription regulatory region DNA binding, calmodulin binding, calcium ion binding, and monooxygenase activity, before and after exposure to chilling stress. This indicated that SlHSP17.7 might exert a substantial effect on transcription and Ca^2+^ signaling (Figure 3B,C).

The enriched Kyoto Encyclopedia of Genes and Genomes (KEGG) pathways of SlHSP17.7 OE showed that photosynthesis-antenna proteins were the top enriched pathways according to the enrichment factor. Moreover, certain genes were involved in metabolic processes, including sphingolipid metabolism, porphyrin and chlorophyll metabolism, glycosphingolipid biosynthesis-ganglio series, glycosphingolipid metabolism, and alpha-linolenic acid metabolism (Figure 3D). After exposure to chilling stress, steroid biosynthesis was the top enriched pathway according to the enrichment factor. Moreover, certain genes were involved in processes such as photosynthesis, linoleic acid metabolism, alpha-linolenic acid metabolism, glycerophospholipid metabolism, and glycerolipid metabolism (Figure 3E).

### 2.4. SlHSP17.7 Overexpression Activated Ca^2+^ Signaling and CBF Transcription Factors under Chilling Stress

**Figure 4:**
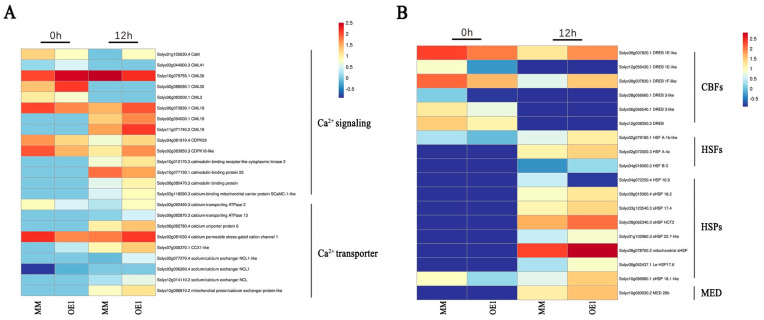
Differential expression of genes involved in transcription factors and signaling. (**A**) The related gene expression of Ca^2+^ signaling in MM and OE1 before and after chilling stress for 12 h. (**B**) The related gene expression of transcription factors and signaling in MM and OE1 before and after chilling stress for 12 h. Red color represents a high expression rank, while blue represents a low expression rank within each individual transcript.

Our previous study showed that SlHSP17.7 interacts with the cation/calcium exchanger 1-like (SlCCX1-like) protein [13]. We performed a virus-induced gene silencing (VIGS) of *SlCCX1-like* in MM and OE1 plants. The phenotypes of the control and the silenced plants under stress are shown in Figure S2. After exposure to 4 °C for seven days, compared with *SlHSP17.7* OE1 plants and empty vector OE1 plants (P-OE1), the gene-silenced plants (V-OE1) showed more wilting under low temperatures. The *SlCCX1-like* VIGS plants showed no resistance to low temperatures. So, the dynamics of SlHSP17.7- and SlCCX1-like plants were consistent. Therefore, we focused on the differential expression of calcium-related signaling pathway genes at the transcriptional level (Figure 4A). Before exposure to chilling stress, some calcium receptors were upregulated, while others were downregulated, but after exposure to chilling stress, most calcium receptors were upregulated, except for *CML36*. Between three calmodulin-binding proteins, two proteins (Solyc10g012170.3, Solyc08g080470.3) were upregulated, and one (Solyc10g077130.1) was downregulated. One calcium-binding protein (Solyc03g118090.3) was upregulated. After exposure to chilling stress, all Ca^2+^ transporters were upregulated. The *SlCCX1-like* (Solyc07g006370.1) gene was significantly upregulated in OE1 at both 0 and 12 h after exposure to chilling stress. This suggested that the OE1 of SlHSP17.7 activated Ca^2+^ signaling and Ca^2+^ transporter under chilling stress.

Calcium signaling affects plant chilling tolerance, mainly through the CBF pathway at low temperatures [22,23]. Before exposure to chilling stress, CBFs of OE1 plants were all downregulated compared to that of MM, while after exposure to chilling stress, two CBFs (Solyc08g007820.1, Solyc08g007830.1) of OE1 plants were upregulated compared to that of MM. Although they (Solyc08g007820.1, Solyc08g007830.1) were downregulated compared to before the exposure to chilling stress, the decrease was much less than that in MM (Figure 4B). Therefore, SlHSP17.7 OE1 maintained CBF signaling under chilling stress.

**Figure 5:**
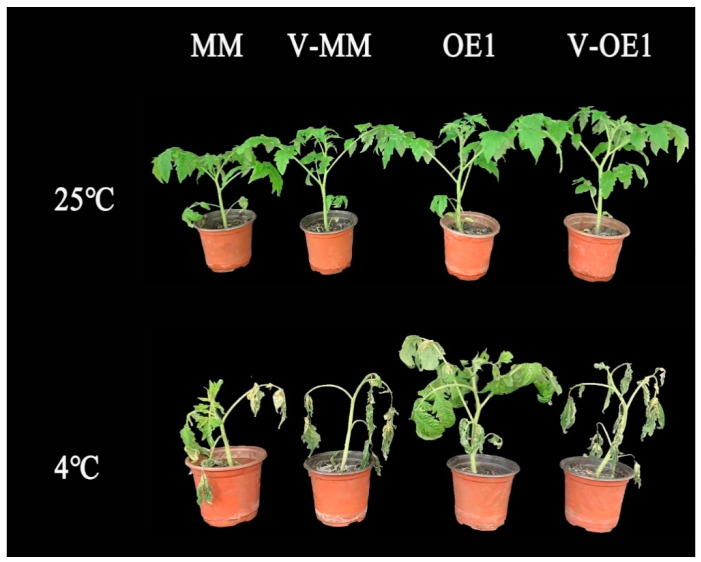
Phenotypic changes of MM, MED26b-silenced MM, OE1, MED26b-silenced OE1 seedlings under chilling stress. MM, control plants; V-MM, MED26b-silenced MM plants; OE1, overexpression of SlHSP17.7 plants; V-OE1, MED26b-silenced OE1 plants.

SlHSP17.7 is involved in a cluster of intronless CI class sHSP genes, whose four members—*SlHSP17.6C* (Solyc06g076570), *SlHSP17.6B* (Solyc06g076560), *SlHSP17.6A* (Solyc06g076540), and *SlHSP17.7A* (Solyc06g076520)—show heterogeneous differential expression profiles during fruit ripening [24]. After silencing, only *SlHSP17.7*-silenced plants suffered the most severe damage under chilling stress [25]. In the transcriptome analysis of OE1 plants, we found that the expression levels of some *sHSPs,* such as *mitochondrial sHSP* (Solyc08g078700.2), *le-HSP17.6* (Solyc08g062437.1), and *sHSP18.1-like* (Solyc10g086680.1), also increased by three to six times compared with those in MM after chilling stress (Figure 4B). This indicated that SlHSP17.7 may have provided a baseline protection in different tomato tissues during chilling stress. From the co-expression network analysis (Table S4) and the transcriptome analysis of *SlHSP17.7* in the plants (Figure S1), we found that the mediator 26b (MED 26b) protein, which was coexpressed with SlHSP17.7, was significantly upregulated after chilling stress (Figure 4B). Consequently, we investigated SlMED26b to further verify the function of its gene through virus-induced gene silencing (VIGS). VIGS vectors PTRV2-SlMED26b were successfully constructed and transformed into Agrobacterium tumefaciens. Tomato seedlings of MM and OE1 plants were injected with Agrobacterium tumefaciens containing the target vector. The expression levels of the target genes in *SlMED26b*-silenced plants and control plants were detected by fluorescence quantitative PCR. Plants with a silencing efficiency greater than 50% compared to the expression level in TRV plants were used for further experiments. We subjected the gene-silenced plants to low-temperature treatment. The phenotypes of the control and silenced plants under stress are shown in Figure 5. After exposure to 4 °C for seven days, compared with the control plants, the gene-silenced plants showed more wilting under low temperatures. Therefore, the overexpression of SlHSP17.7 increased cold tolerance in transgenic tomato plants, whereas the virus-induced gene-silencing-mediated suppression of SlMED26 increased cold sensitivity in the seedlings of tomato.

### 2.5. SlHSP17.7 Overexpression Positively Regulated Reactive Oxygen Species (ROS)-Scavenging-Related Genes and Alleviated ROS Accumulation under Chilling Stress

**Figure 6:**
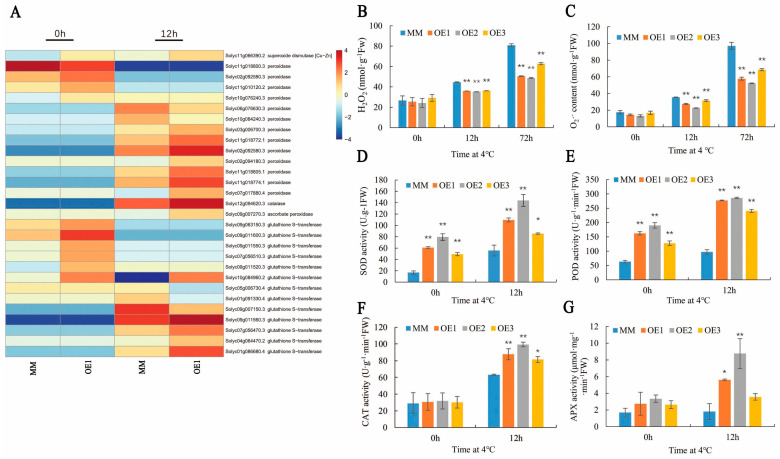
SlHSP17.7 alleviates ROS accumulation. (**A**) ROS-scavenging-related genes differentially expressed between MM and OE1 before and after chilling stress for 12 h. (**B**) H_2_O_2_ content of MM and OE after chilling stress for 12 h and 72 h. (**C**) O_2_·^−^ content of MM and OE after chilling stress for 12 h and 72 h. (**D**) SOD activity of MM and OE before and after chilling stress for 12 h. (**E**) POD activity of MM and OE before and after chilling stress for 12 h. (**F**) CAT activity of MM and OE before and after chilling stress for 12 h. (**G**) APX activity of MM and OE before and after chilling stress for 12h. For (B−G), error bars represent the SD with three biological replicates (*, *p* < 0.05; **, *p* < 0.01).

According to the transcriptome analysis of OE1 plants, numerous ROS-metabolism-related enzyme genes were differentially expressed. Under normal conditions, the transcriptional levels of *Cu/Zn SOD, PODs,* and *GSTs* in the OE1 lines were much higher than those in the MM plants except for one *POD* (Solyc11g018800.3). After exposure to chilling stress, the expressions of “ROS scavenging enzyme” genes increased. In the OE1 lines, *Cu/ZnSOD*, catalase (*CAT*), and ascorbate peroxidase (*APX*) were highly expressed. Seven out of nine PODs were highly expressed except for two *PODs* (Solyc06g076630.3 and Solyc10g084240.3), and five out of eight *GSTs* were highly expressed except for three *GSTs* (Solyc05g006730.4, Solyc01g091330.4, and Solyc09g007150.3) (Figure 6A).

The primary consequence of low-temperature stress is the excessive accumulation of ROS due to the disrupted electron transport chain in chloroplasts. To investigate the alleviating effect of *SlHSP17.7* overexpression on low-temperature-induced oxidative stress, we quantified the H_2_O_2_ content and production of O_2_·^–^ in OE plants under chilling stress. It was observed that chilling stress led to a dramatic increase in the level of H_2_O_2_ and O_2_·^–^ in MM plants, while the OE lines (OE1, OE2, and OE3) showed a reduced accumulation of H_2_O_2_ and O_2_·^–^ (Figure 6B,C). The H_2_O_2_ content of OE1, OE2, and OE3 plants decreased by 20%, 21%, and 19% and 37%, 40%, and 22% compared to MM plants after exposure to chilling stress for 12 and 72 h, respectively. The O_2_·^–^ content of OE1, OE2, and OE3 plants decreased by 35%, 36%, 11% and 40%, 46%, and 29% compared to MM plants after exposure to chilling stress for 12 and 72 h, respectively. These results indicate that *SlHSP17.7* OE might favor the removal of the chilling-induced excessive production of ROS in tomato leaves.

Plants have developed a sophisticated antioxidant system to cope with oxidative stress. To investigate the impact of *SlHSP17.7* OE on the antioxidant system, we assayed the activities of typical antioxidant enzymes. Under normal growth conditions, the activities of CAT and APX in overexpressed lines were not significantly different from those in MM, unlike the activities of SOD and POD in overexpressed lines, which were significantly higher than those in MM. After exposure to chilling stress for 12 h, the activities of four enzymes were significantly enhanced in all OE plants than in MM plants. Thus, the results were consistent with the transcriptome data (Figure 6D–G). This suggests that SlHSP17.7 increased chilling tolerance in tomato plants by regulating these ROS scavenging genes.

### 2.6. SlHSP17.7 Overexpression Promoted Starch Formation under Normal Conditions and Accumulates More Soluble Sugars under Chilling Stress

**Figure 7:**
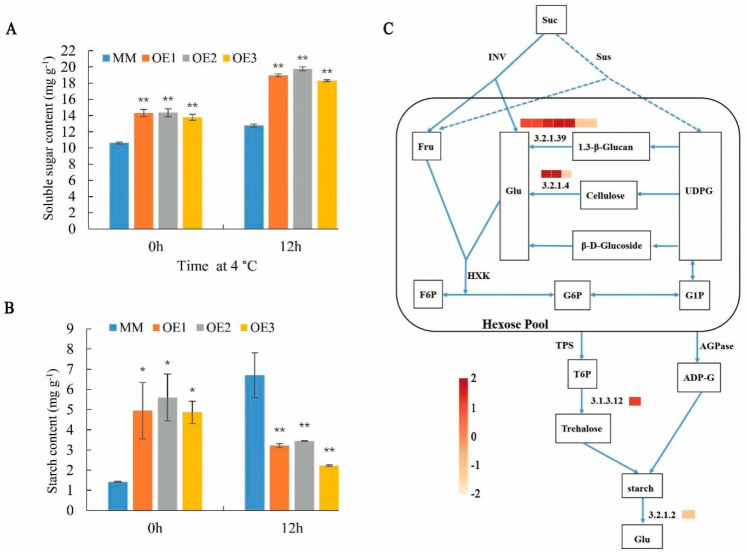
SlHSP17.7 promotes starch formation under normal conditions. (**A**) Determination of soluble sugar content. (**B**) Determination of starch content. For (**A**,**B**), Error bars represent the SD with three biological replicates (*, *p* < 0.05; **, *p* < 0.01). (**C**) Pathway diagram of starch synthesis and decomposition. FPKM values are used in the heat maps (MM-0 h vs. OE-0 h). Red color represents upregulated gene, while orange represents downregulated gene. Abbreviations: Suc, sucrose; Sus, sucrose synthase; INV, invertase; Fru, fructose; Glc, glucose; UDPG, uridine-diphosphoglucose; HXK, hexokinase; F6P, fructose-6-phosphate; G6P, glucose-6-phosphate; G1P, glucose-1-phosphate; TPS, trehalose-6-phosphate synthase; T6P, trehalose-6-phosphate; AGPase, ADP-glucose pyrophosphorylase; ADP-G, ADP-glucose. Arrows (→) signify the direction or stimulation of the reaction.

The results show that the overexpression of SlHSP17.7 genes significantly influenced the accumulation of carbohydrate in the leaves. The soluble sugar contents of the OE1, OE2, and OE3 were 1.35, 1.35 and 1.30 times that of MM, respectively (Figure 7A). The starch contents of the OE1, OE2, and OE3 were significantly higher than those of the control, and in the mature leaves, the starch contents were 3.51, 3.97, 3.45 times that of MM, respectively (Figure 7B). The electron microscopy results indicate that the amount of starch formed by the OE1 plants was much higher than that in the MM plants. To investigate whether the altered accumulation of carbohydrate was related to SlHSP17.7 OE, we assessed the transcript levels of genes involved in carbohydrate biosynthesis or metabolism in 6-week-old leaves (Figure 7C). In agreement, we detected higher transcript levels for starch biosynthesis genes (five beta-glucosidase genes (EC 3.2.1.39), two endoglucanase genes (EC 3.2.1.4), and trehalose-6-phosphate phosphatase (TPP; EC 3.1.3.12)). At the same time, beta-amylase (EC 3.2.1.2), which belongs to the starch degradation genes, was downregulated. Therefore, the amounts of starch were increased in the OE plants.

Following exposure at 4 °C for 12 h, starch accumulation in the OE lines considerably decreased. The starch content of the OE1, OE2, and OE3 decreased to 52%, 49%, 67% compared to MM, respectively (Figure 7B). Moreover, the soluble sugar increased compared with those in the MM plants, the soluble sugar content of the OE1, OE2, and OE3 were 1.49, 1.55, 1.43 times those of MM, respectively (Figure 7A). Taken together, these results indicate that, in addition to its role in the accumulation of carbohydrate, SlHSP17.7 also participates in tolerance to chilling stress, at least partly through the alteration of carbohydrate production.

### 2.7. SlHSP17.7 Overexpression Improved the Fluidity of Cell Membrane and Phosphatidylglycerol (PG) Metabolism upon Metabolite Identification

To verify how the overexpression of *SlHSP17.7* changed the activity of photosystem, we performed metabolome analysis using OE1 plants. All metabolites were classified into four categories according to the KEGG databases. The “metabolite” was the largest term, while the top seven pathways were “global and overview maps,” “biosynthesis of other secondary metabolites,” “amino acid metabolism,” “metabolism of terpenoids and polyketides,” “carbohydrate metabolism,” “metabolism of cofactors and vitamins,” and “lipid metabolism” in both positive (POS) and negative (NEG) models (Figure S3A,B).

All 22,371 and 19,840 metabolites identified were classified into 24 and 22 HMDB super classes in POS and NEG models, respectively. Between them, 10,329 and 9798 metabolites were included in “lipids and lipid-like molecules,” which was the largest class in both POS and NEG models (Figure S3C,D).

In the lipid metabolism of OE1, several polyunsaturated fatty acids metabolites included in “linoleic acid metabolism” (Figure S3A,B), “α−linolenic acid metabolism” (Figure S5A,B), and “arachidonic acid metabolism” (Figure S6A,B) were upregulated before and after exposure to chilling stress. As an important component of membrane phospholipids, polyunsaturated fatty acids can enter the lipid bilayer of membranes, affecting their fluidity. We also found that most fatty acids were increased before or after chilling stress for 12 h. Ten fatty acids increased by 2–3-fold, two fatty acids (M145T53; M337T316) were decreased by −0.36-fold and −0.23-fold, respectively (Table 1). Concurrently, some glycerophospholipids and glycolipids were altered under chilling stress, especially, PG32:1 (16:0/16:1), containing 16:0 at the sn-1 and t16:1 at the sn-2. PG32:1 showed a higher abundance with a 7.08- and 4.31-fold increase at 0 and 12 h, respectively. These results indicate that SlHSP17.7 may enhance the low-temperature tolerance and the activity of the photosystem by affecting lipid metabolism to protect membranes fluidity and stability.

## 3. Discussion

In our previous study, we showed that SlHSP17.7 was localized in the cytoplasm and improved the chilling tolerance of tomato fruits [8]. In this study, we analyzed the roles of SlHSP17.7 in tomato seedlings in response to low temperatures, making use of SlHSP17.7 OE of T4 generation. Our results show that the overexpression of *SlHSP17.7* enhanced the chilling tolerance of tomato seedlings (Figure 1). Compared with MM plants, the OE plants showed decreased REC, MDA, H_2_O_2_, and O_2_·^–^ content after exposure to chilling stress.

Temperature is a physical signal, and it can be sensed by plants through the plasma membrane [26]. Low temperatures can reduce cell membrane fluidity [27]. Polyunsaturated fatty acids, important components of membrane phospholipids, can enter the lipid bilayer of membranes, thereby affecting their fluidity and stability and playing an important role in determining the function of the membrane [28]. In this study, in plants with *SlHSP17.7* OE1, many metabolites included in “alpha-linolenic acid metabolism,” “linoleic acid metabolism,” and “arachidonic acid metabolism” were upregulated before and after exposure to chilling stress for 12 h (Figures S4–S6). Furthermore, most fatty acids were also upregulated before and after exposure to chilling stress for 12 h (Table 1). Increased polyunsaturated fatty acids content could reduce the melting point of membrane lipids and enhance membrane fluidity [29]. Polyunsaturated lipids maintain cellular function and meet physiological needs at low temperatures [30]. The photosynthetic ability of the OE plants was less damaged, as reflected in the higher chlorophyll content, Pn, Fv/Fm, and φPSII compared with MM plants after exposure to chilling stress. The chloroplast ultrastructure showed that the thylakoid stacking of *SlHSP17.7* OE1 plants was thicker than that in the MM plants, while the chloroplast was less impaired after exposure to chilling stress. Hence, plants with *SlHSP17.7* OE maintained membrane fluidity under chilling stress.

sHSPs can modulate membrane lipid polymorphism, fluidity, and the composition of cell membranes [31,32]. In this study, in OE1 plants, PG32:1 (16:0/16:1) significantly increased before and after exposure to chilling stress. PG is indispensable for forming LHCII trimer and thylakoid stacking [33,34]. PG of the thylakoid membrane containing trans-16:1 was important for the stabilization of the oligomeric states of PSII and its light harvesting systems [35]. Trans-16:1 abounds in the PG molecule of the thylakoid membrane but is absent in other lipids. As observed from cyanobacteria mutants lacking PG synthesis, PG was deemed necessary for normal cell growth and helped maintain the structure of the QB-binding site in the D1 protein [36,37,38]. Furthermore, PG is crucial in thylakoid membrane development in *Arabidopsis thaliana* and affected photosynthesis [39,40]. The PSII of cucumber is rich in PG, and the trans-16:1 in the PG molecule helps maintain the structure of LHCII oligosaccharide. When the content of trans-16:1 is low, LHCII oligomers decreased, while monomers increased. Furthermore, the energy captured by LHCII could not be effectively transferred to the reaction center, and the light capture efficiency decreased [41,42]. Hence, PG might assist the development of chloroplasts and the stabilization of photosynthetic machinery [43]. PG32:1 (16:0/16:1) showed higher abundance with a 7.08-fold increase in OE1 plant compared with MM. The chloroplast ultrastructure showed that the thylakoid stacking of the *SlHSP17.7* OE1 was also thicker than that in the MM plants. Thereby, the increase in phosphatidylglycerol (PG) content promoted the formation of thylakoid stacking. This may be the reason for the higher net photosynthetic rate in OE plants. The content of trans-16:1 is easily affected by external conditions such as temperature and illumination; PG34:4 (18:3/16:1) in wheat showed a significant decrease after low-temperature acclimation [44], suggesting that environmental factors can change the structure of LHCII by changing the content of trans-16:1, ultimately changing the photosynthetic activity. Therefore, SlHSP17.7 may enhance low-temperature tolerance in tomato plants by increasing the content of PG32:1 (16:0/16:1), thereby promoting the formation of thylakoid stacking and photosynthesis.

Calcium is an essential nutrient in plants and participates in regulating many physiological processes as a second messenger. At the same time, Ca^2+^ is also involved in the chilling stress-specific signal transduction [45,46]. When plants are subjected to low-temperature stress, Ca^2+^ channels are activated, intracellular Ca^2+^ concentration increases, and specific calcium signals are generated. These are then transmitted to calcium receptors, thus activating the expression of downstream early-response genes and triggering the plant response to low-temperature stress [47,48]. In this study, *SlCCX1-like* genes were significantly upregulated in OE1 plants at both 0 and 12 h after chilling stress, which might increase intracellular calcium concentration (Figure S7). At the same time, calcium receptors, including calmodulin (CaM), Ca^2+^-dependent protein kinases (CDPKs), and CaM-like proteins (CML), were virtually upregulated after exposure to chilling stress (Figure 3A). Calcium-dependent protein kinases (CDPKs) were involved in the activation of mitogen-activated protein kinase (MAPK) cascades [49], suggesting that the OE1 of *SlHSP17.7* accelerated the expression of calcium transporter protein and activated Ca^2+^ signaling under chilling stress.

The overexpression of the core binding factor (CBF) (C-repeat binding transcription factor/dehydrate responsive element binding factor, DREB) can regulate the expression of downstream cold-resistant genes and is extremely important for plants’ resistance to low temperatures [50], at which Ca^2+^ is influxed into the cytoplasm through Ca^2+^ channels, while Ca^2+^ signaling induces the expression of CBF/COR genes [51,52]. Several CBF target genes, including ROS detoxification and stress response genes, have been identified in CBF-OE plants [53]. The heterologous expression *AtCBF1* [54,55,56], *SlGRAS4* [57], or *LimHSP16.45* [58] can all protect plants against abiotic stresses by increasing antioxidant capacity to scavenge cellular reactive oxygen species. In this study, Ca^2+^ signaling, CBF transcription factors, antioxidant enzymes, and antioxidant enzyme-related genes were upregulated in SlHSP17.7-OE plants. These results suggest that Ca^2+^ signaling induced the expression of CBF genes and CBF inhibited the production of ROS.

Interestingly, while exploring the role of SlHSP17.7 before and after exposure to chilling stress [8], we observed that SlHSP17.7 interacted with SlCCX1-like, and their expression levels were negatively correlated in the tomato fruit. However, in leaves, their expression levels synergistically change after exposure to chilling stress. The possible reason for this is that SlHSP17.7 forms different complexes in leaves and fruit as chaperones and shows different gene expressions such as human Hsp90 cochaperones [59].

Mediators are involved in the transcriptional regulation of RNA polymerase II (Pol II) by acting as coregulators [60]. They interact with gene-specific transcription factors [61] and modulate several signaling pathways such as cell differentiation, cell growth, and tissue development [60,62]. MED16, MED14, and MED2 regulate mediators and RNA polymerase II recruitment to CBF-responsive cold-regulated genes in *Arabidopsis*. In addition, these three subunits are required for the expression of certain other cold-responsive genes [63]. The gene coexpression network (http://genemania.org/ accessed on 17 January 2022) analysis shows that SlHSP17.7 is coexpressed with MED (Table S4). In the transcriptome, we excavated the MED26b subunit whose expression level was significantly increased in *SlHSP17.7* OE under chilling stress, and after MED26b silencing, the plants exhibited a significantly lower temperature sensitivity. Therefore, MED26b may promote the transcription of *SlCCX1-like*, *CBF*, and genes associated with lipid metabolism to enhance chilling tolerance. However, how MED co-expresses with HSP to regulate gene transcription remains unclear and requires further experimental analysis. Moreover, the response of plants to low temperature is a process of gradual adaptation, including transcription, metabolism, and its related physiological processes. These processes that follow cold adaptation require further experimental investigation.

## 4. Materials and Methods

### 4.1. Materials and Chilling Stress Treatments

Wild-type tomatoes (*Solanum lycopersicum* L. cv. Moneymaker) and three *SlHSP17.7* OE T4 transgenic lines (OE1, OE2, and OE3) were used in this study [13]. When the tomato seedlings grew two leaves, we transferred them into a nutrition bowl and placed them in a growth chamber with a regime of 25 °C/18 °C (day/night), 16 h/8 h (light/dark) photo-period, and a relative humidity (RH) of 60–70%. The photon flux density (PFD) was 500 μmol m^−2^ s^−1^.

Four-week-old plants at the six-leaf stage were incubated in a 4 °C HDL brand HPG-280BX type artificial climate box with light. Each treatment group included five seedings and the process was repeated three times. The PFD was 200 μmol m^−2^ s^−1^; the illumination time was 12 h; RH was 60–70%. The second set of fully expanded leaves of OE1 was collected at 0 and 12 h after exposure to chilling stress, frozen in liquid nitrogen, stored at −80° C, and later used for the extraction of total ribonucleic acid (RNA) for transcriptomic analysis. The phenotype of the tomato seedlings was observed after 7 d of cold stress.

### 4.2. Evaluation of Chilling Injury Index

The six-leaf stage tomato seedlings at 4 °C grown for 7 d were used for the evaluation of chilling injury (CI). The treatment condition was the same as described above. Each treatment group (MM, OE1, OE2, and OE3) included three seedlings and the process was repeated three times. The classification standard and CI index were calculated according to the method of Chen et al., (2019) [64] as follows: 0 = no injury; 1 = leaves with a few scattered pits and margins that turned yellow; 2 = leaf discoloration and wilting; 3 = leaves wilt and petioles slightly droop; 4 = leaves decay and petioles seriously droop; 5 = lodging of the whole plant. The CI index was recorded with the following formula:

$$\mathrm{CI}\;\mathrm{index}\;(\mathrm{between}\;0\;\mathrm{and}\;5)=\frac{\sum \left[(\mathrm{CI}\;\mathrm{Level})\;\times \;(\mathrm{Number}\;\mathrm{of}\;\mathrm{plants}\;\mathrm{at}\;\mathrm{the}\;\mathrm{CI}\;\mathrm{level})\right]}{\mathrm{Total}\;\mathrm{number}\;\mathrm{of}\;\mathrm{plants}\;\mathrm{in}\;\mathrm{the}\;\mathrm{treatment}}$$

### 4.3. Measurement of Physiological Parameters

When the tomato seedlings grew eight leaves, the four fully unfolded leaves from the top were selected to measure the net photosynthetic rate (Pn). The Pn was measured using LI-6800 (LI-COR, Lincoln, NE, USA) at 25 °C with an RH of 60%, CO_2_ of 400 μmol·mol^−1^, and a photon flux density (PFD) of 600 μmol·m^−2^·s^−1^. Before measuring, the tomato seedlings were kept at 25 °C at a PFD of 100 μmol·m^−2^·s^−1^ for 30 min to induce stomatal opening and illuminated at a PFD of 600 μmol·m^−2^·s^−1^ for 15 min. The φPSII and Fv/Fm were also measured using LI-6800 (LI-COR, USA) following the manufacturer’s recommendations. Before φPSII measurement, the plants were kept for at least 20 min at a PFD of 600 μmol·m^−2^·s^−1^. Before measuring the Fv/Fm, the tomato seedlings were kept in the dark for at least 30 min. Chlorophyll was extracted using 95% ethanol and analyzed using ultraviolet (UV) spectrophotometry [65].

The frozen leaves (0.5 g) were extracted in a 5 mL 50 mM potassium phosphate buffer at pH 7.8, which contained 1 mM ethylenediaminetetraacetic acid (EDTA) and 1% polyvinylpyrrolidone and were ground on ice. The homogenate was centrifuged at 13,000× *g* for 20 min at 4 °C and the supernatant was used for the subsequent antioxidant enzyme assays. The activities of superoxide dismutase (SOD), catalase (CAT), and ascorbate peroxidase (APX) were assessed according to the methods of Jiang et al. [66] and Zhang et al. [67]. Moreover, the activity of peroxidase (POD) was measured following the method described by Rao et al. [68].

For the measurement of malondialdehyde (MDA) content, 2.5 mL of 0.5% thiobarbituric acid reagent (20% trichloroacetic acid containing 0.5% thiobarbituric acid) was added to 1.5 mL of the above enzyme solution, heated at 100 °C for 30 min, and cooled on ice. The supernatant was centrifuged at 1800× *g* for 10 min. The absorbance was recorded at 532 nm and 600 nm.

The relative electrical conductivity (REC) and O_2_·^–^ were determined as described by Kong et al. [69]. However, the methods used for the measurement of O_2_·^–^ had a slight change. The leaves (0.2 g) were ground with 3 mL cold phosphate buffer (50 mM, pH 7.8) and transferred into a centrifugal tube. The homogenate was centrifuged for 20 min at 15,000× *g* and 4 °C. We added phosphate buffer (pH 7.8) and 10 mM hydroxylammonium chloride to the supernatant before the first exposure to 25 °C for 20 min; thereafter, 58 mM sulfanilic amide and 7 mM α-naphthylamine were added and the mixture was incubated for 20 min at 25 °C; the same volume of trichloroacetic acid was then added. Finally, the mixture was centrifuged at 15,000× *g* for 1 min. The absorbance at 530 nm was recorded for the supernatant.

H_2_O_2_ concentration was measured following the method of Ibrahim et al. [70]. The leaves (0.3 g) were ground with 0.1% trichloroacetic acid (TCA; 5 mL) in a mortar and centrifuged for 15 min at 15,000× *g* and 4 °C. Thereafter, 10 mM phosphate buffer (0.5 mL) and 1 M KI (1 mL) were added to the supernatant (1 mL). The absorbance was measured at 390 nm.

The total soluble sugar was extracted with 80% ethanol from leaves (0.5 g) [71], while the insoluble residue mixed with 9.2 mol·L^−1^ perchloric acid was used for starch extraction [72,73]. The soluble sugar and the starch were all estimated using the anthrone colorimetric method at 620 nm and 625 nm, respectively, using glucose as the standard.

### 4.4. Observation of Chloroplast Ultrastructure

For transmission electron microscopy, mesophyll tissues in the leaves of MM and OE1 plants were collected before and after chilling stress for 12 h and fixed in 2.5% glutaraldehyde at 4 °C for 7 d. The sample was rinsed with phosphoric acid buffer (pH 7.2–7.4) and soaked for 15 min; the process was repeated three times. Subsequently, 1% osmium acid was used for 2 h post-fixation. The leaves were dehydrated through a series of ethanol and dehydrated twice with 100% acetone. The leaves were infiltrated and embedded using epoxy resin; subsequently, 70–90 nm of ultrathin sections was cut with an ultramicrotome and stained using uranyl acetate and lead citrate. Finally, the observation and imaging of stained sections were performed using a Hitachi-7700 transmission electron microscope (Hitachi, Tokyo, Japan).

### 4.5. RNA Sequencing Analysis

For RNA sequencing (RNA-seq), total RNAs were extracted from MM and OE1 plants incubated at 4 °C for 0 and 12 h. After the total RNA was qualified, eukaryotic mRNA was enriched by magnetic beads connected with Oligo(dT). The extracted mRNA was fragmented into short fragments using the fragmentation buffer, and a cDNA strand was synthesized with random hexamers using fragment mRNA as template. Twelve cDNA libraries were constructed and sequenced (three biological replicates) using an Illumina Novaseq™ 6000 at the LC-BIO (Hangzhou, China). The reads with adaptors or unknown nucleotides > 5%, and low-quality reads with more than 20% Q ≤ 10 bases were discarded. Gene expression analysis was performed using the method of fragments per kilobase of exon model per million mapped reads (FPKM), and criteria of q < 0.05 and log2 fold- change (log2FC) ≥ 1 were used for determining differentially expressed genes (DEGs). For gene ontology (GO) terms and KEGG pathway enrichment analysis, a cutoff of *p* < 0.05 was established.

### 4.6. qRT-PCR Analysis

The total RNA was extracted using TRNzol Universal Reagent (Tiangen, Beijing, China) from the MM and OE plants incubated at 4 °C for 12 h. A strand of cDNA was synthesized from mRNA using PrimeScriptTMRT reagent Kit (Takara, Dalian, China) following the manufacturer’s instructions. Quantitative PCR reactions were performed in triplicate with the SYBR qPCR master mix (Vazyme, Nanjing, China) following Vazyme’s recommendations. qRT-PCR was conducted on a Bio-Rad CFX manager 3.1 real-time PCR instrument (Bio-Rad, Hercules, CA, USA). The fold change was calculated by means of the 2-ΔΔCT method, and actin was used as the quantitative control. All primers utilized for the SlHSP17.7 expression analysis are listed in Table S1.

### 4.7. Cloning MED26b and VIGS Vector Construction

Total RNA isolation and cDNA synthesis were performed as previously described [74]. The MED26b fragments (Solyc10g080930.2) were amplified from the cDNA of Moneymaker leaves via specific primers MED vigs-F and MED vigs-R (Table S1). The PCR product and pTRV2 vector, which were digested by BamHI and XhoI restriction enzymes and recovered were ligated with T4 DNA ligase enzyme (Takara, Beijing) overnight at 16 °C and transformed into E. coli DH5α. The recombinant of pTRV2-MED26b was verified using PCR with a bacterial solution using the primers vigs-F and vigs-R (Table S1).

### 4.8. Agrobacterium Preparation and Inoculation

The plasmids pTRV1, pTRV2, or pTRV2- MED26b were transformed into *Agrobacterium* strain GV3101, separately. The transformed cells were grown in Luria-Bertani (LB) medium (50 mg/L kanamycin and 50 mg/L rifampicin, 10 mM MES, 20 μM acetosyringone) at 28 °C overnight. Three *Agrobacterium* cells were collected and suspended in injection buffer (10 mM MgCl_2_, 10 mM MES, 200 μM acetosyringone) with an OD_600_ of 1.0. *Agrobacterium* cultures containing pTRV1 and pTRV2 or pTRV2-MED26b were mixed at the ratio of 1:1 before inoculation.

The seeds were soaked for 20 min at 55 °C, placed in conical jars, and incubated at 28 °C for 2–3 days. Germinated or non-germinated seeds were placed in *Agrobacterium* solution and vacuumized, such that the solution soaked into the plant tissue. The germinated or ungerminated seeds were then planted in the ground and cultured at 25 °C and 12 h/12 h (light/dark) conditions. Three replicates were set for each treatment, and 30 seedlings were set for each replicate. qRT-PCR was used to further validate the efficiency of silencing at the four leaves stage. The primers used are listed in Table S1. The conditions of qRT-PCR were as follows: one cycle of denaturation (95° C, 30 s); 40 amplification cycles (95 °C, 10 s; 55 °C 30 s), and a signal acquisition (65° C, 5 s).

### 4.9. Metabolite Extraction and Analysis

The frozen leaves (0.1g) were ground with liquid nitrogen and extracted in 120 µL of 50% methanol. Later, the mixture was vortexed for 1 min, incubated for 10 min under room temperature, and preserved at −20 °C overnight. The mixture obtained was centrifuged at 4000× *g* for 20 min, and the supernatant was transferred to a new 96-well plate. A pooled quality-control (QC) sample was prepared by combining 10 μL of each extraction mixture.

All samples were analyzed using a liquid chromatography–mass spectrometry (LC-MS) system. First, an ultra-performance liquid chromatography (UPLC) system (SCIEX, Macclesfield, UK) was used for chromatographic separations. Then, reversed phase separation was performed on the ACQUITY UPLC T3 column (100 mm × 2.1 mm, 1.8 µm, Waters, Wilmslow, UK). The column oven was kept at 35 °C. Solvent A (water, 0.1% formic acid) and solvent B (acetonitrile, 0.1% formic acid) were the mobile phases, and the flow rate was 0.4 mL·mL^−1^. The gradient elution conditions were set as follows: 0–0.5 min, 5% B; 0.5–7 min, 5% to 100% B; 7–8 min, 100% B; 8–8.1 min, 100% to 5% B; 8.1–10 min, 5% B. The injection volume for each sample was 4 µL.

The Triple TOF 5600 Plus system was used to detect metabolites. The raw data were converted to mzXML and processed using the XCMS, CAMERA and metaX software. Comprehensive information on retention time and m/z were used to identify each ion. KEGG (http://www.kegg.jp/), in-house (http://spldatabase.saskatoonlibrary.ca/), and HMDB (http://www.hmdb.ca/) databases were used to explain the properties and functions of the metabolites. The differential metabolites were quantified and screened using the metaX software (http://metax.genomics.cn/).

### 4.10. Data Analysis and Statistics

For each experiment, three biological replicates and three technical replicates were used. The significant differences among the treatments and the control were determined according to the one-way ANOVA test using the SPSS Statistics 17.0 software (IBM Corp., Armonk, NY, USA). The standard errors were calculated and showed as error bars. * and ** indicate significant and highly significant differences between the treatments and the control at *p*-values ≤ 0.05 and 0.01, respectively.

## 5. Conclusions

**Figure 8:**
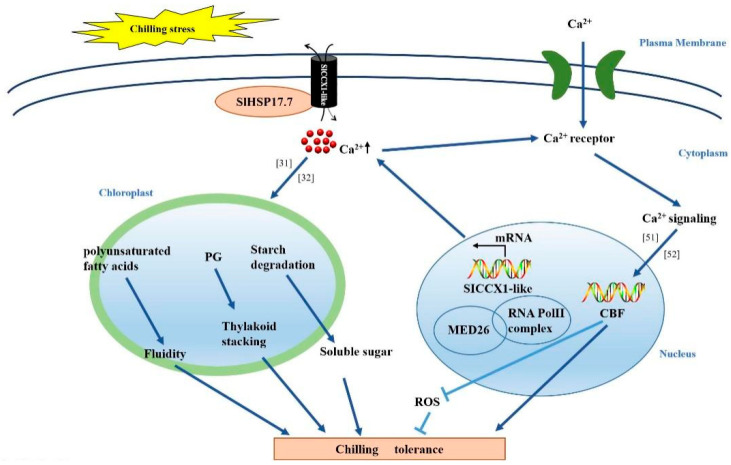
Proposed model for chilling tolerance in *SlHSP17.7* OE tomato plants. SlHSP17.7 can act on the cell membrane, improve membrane fluidity by increasing the content of polyunsaturated fatty acids. The increase in PG contents promotes the formation of thylakoid stacking. SlHSP17.7 also decreases chilling-induced starch accumulation and increases soluble sugar content. SlHSP17.7 interacted with SlCCX1-like genes, intracellular calcium concentration increased, calcium receptors were activated. Ca^2+^ signaling induce the expression of CBF genes, CBF inhibit the production of ROS. MED26b, which was coexpressed with SlHSP17.7, may promote the transcription of the related genes. So, SlHSP17.7 enhances the resistance of tomato plants to chilling stress. Arrowheads and end lines indicate positive and negative regulation, respectively. The literature is noted as follows: [31,32,51,52].

In summary, our observations present a strong evidence to support the multiple roles of SlHSP17.7, such as the regulation of membrane integrity and the interaction with SlCCX1-like to modulate plant responses to chilling (Figure 8). SlHSP17.7 can act on the cell membrane to improve membrane fluidity by increasing the contents of polyunsaturated fatty acids in the membrane and indirectly protects the chloroplast by increasing the content of PG [31,32]. SlHSP17.7 also decreased chilling-induced starch accumulation and increased soluble sugar content. Cytoplasm-localized SlHSP17.7 interacted with SlCCX1-like genes to increase intracellular Ca^2+^ concentration, activate Ca^2+^ signaling, and induce the expression of CBF genes under chilling stress [51,52]. MED26b, coexpressed with SlHSP17.7, may promote the transcription of the related genes. Above all, SlHSP17.7 improved chilling tolerance in tomato plants. Our results provide novel insights into improving the yield production and the quality of tomato plants and may have implications in other crops.

## Figures and Tables

**Table 1:** The fold changes of differential metabolites in lipid metabolism.

Class	ID	MS2 Metabolite	Log_2_FC (0 h)	Log_2_FC (12 h)
Glycerophospholipids	M719T496	PG 32:1; PG (16:0/16:1)	+7.08	+4.31
	M518T391	LysoPS 18:3; LysoPS 18:3		+3.34
	M571T536_2	LysoPI 16:0; LysoPI 16:0		−0.44
	M695T527	PA 36:4; PA (18:2/18:2)	−0.32	
	M781T462	1-Palmitoyl-2-linoleoyl-sn-glycero-3-phosphocholine	−0.04	
	M781T493	1-Palmitoyl-2-linoleoyl-sn-glycero-3-phosphocholine	−0.08	
	M781T533	1-Palmitoyl-2-linoleoyl-sn-glycero-3-phosphocholine	−0.21	
	M184T59	Phosphocholine	−0.08	
Glycerolipids	M815T497	SQDG 34:3; SQDG (16:0/18:3)	−0.45	
	M830T521	SQDG 35:3; SQDG (17:0/18:3)	−0.23	−0.33
Fatty acids	M279T422	2-2-Hexyldecanoic acid	+2.13	
	M293T414	9-Oxo-10E,12Z,15Z-octadecatrienoic acid	+2.02	
	M328T288	9-Oxo-11-(3-pentyl-2-oxiranyl)-10E-undecenoic acid		+2.41
	M328T346	9-Oxo-11-(3-pentyl-2-oxiranyl)-10E-undecenoic acid		+2.44
	M265T377	Dinor-12-oxophytodienoic acid		+2.12
	M353T393	Monolinolenin (9c,12c,15c)		+2.26
	M145T53	(E)-2-Methylglutaconic acid		−0.36
	M337T316	8,11-Tridecadienoic acid, 13-(3-pentyl-2-oxiranyl)-, methyl ester, (8Z,11Z)-		−0.23
	M221T231	2,3-Dimethyl-3-hydroxyglutaric acid		+3.08
	M293T400_2	9-OxoODE		+2.00
	M311T353	(E)-13-Hydroxy-10-oxo-11-octadecenoic acid	+2.53	
	M275T400	Stearidonic acid	+2.35	

Note: The positive number indicates an increase and negative numbers indicate a decrease; 0 h and 12 h represent before and after chilling stress for 12 h.

## Data Availability

Not applicable.

## Supplementary Materials

The following supporting information can be downloaded at: https://www.mdpi.com/article/10.3390/plants11141865/s1, Figure S1: Verifification of RNA-seq results by qRT-PCR (MM-12h vs OE-12h); Figure S2: Phenotypic changes of MM, SlCCX1-like-silenced MM, OE1, SlCCX1-like-silenced OE1 seedlings under chilling stress; Figure S3 Metabolite identification. A. MS1-KEGG/pos; B. MS1-KEGG/neg; C. MS1-HMDB/pos; D. MS1-HMDB/neg; Figure S4: The differential metabolites in linoleic acid metabolism before (A) and after chilling stress for 12 h (B); Figure S5: The differential metabolites in alpha-linolenic acid metabolism before (A) and after chilling stress for 12 h (B); Figure S6: The differential metabolites in arachidonic acid metabolism before (A) and after chilling stress for 12 h (B); Figure S7: Overexpression of SlCCX1-like in Micro-Tom (CK) increased calcium concentration of the leaves; Table S1: List of primers used in this paper; Table S2: Differentially expressed genes (DEGs) of photosynthesis before chilling stress; Table S3: Differentially expressed genes (DEGs) of photosynthesis after chilling stress for 12h; Table S4: List of co-expressed MED and HSP genes.
