# Supplementary material for: SlHSP17.7 Ameliorates Chilling Stress-Induced Damage by Regulating Phosphatidylglycerol Metabolism and Calcium Signal in Tomato Plants
Source: Plants (Basel). 2022 Jul 18;11(14):1865. doi: 10.3390/plants11141865 (PMC9324031; doi:10.3390/plants11141865)
Supplement: Supplementary file 1 [file plants-11-01865-s001.zip › Figure S-HSP17.7.pdf]

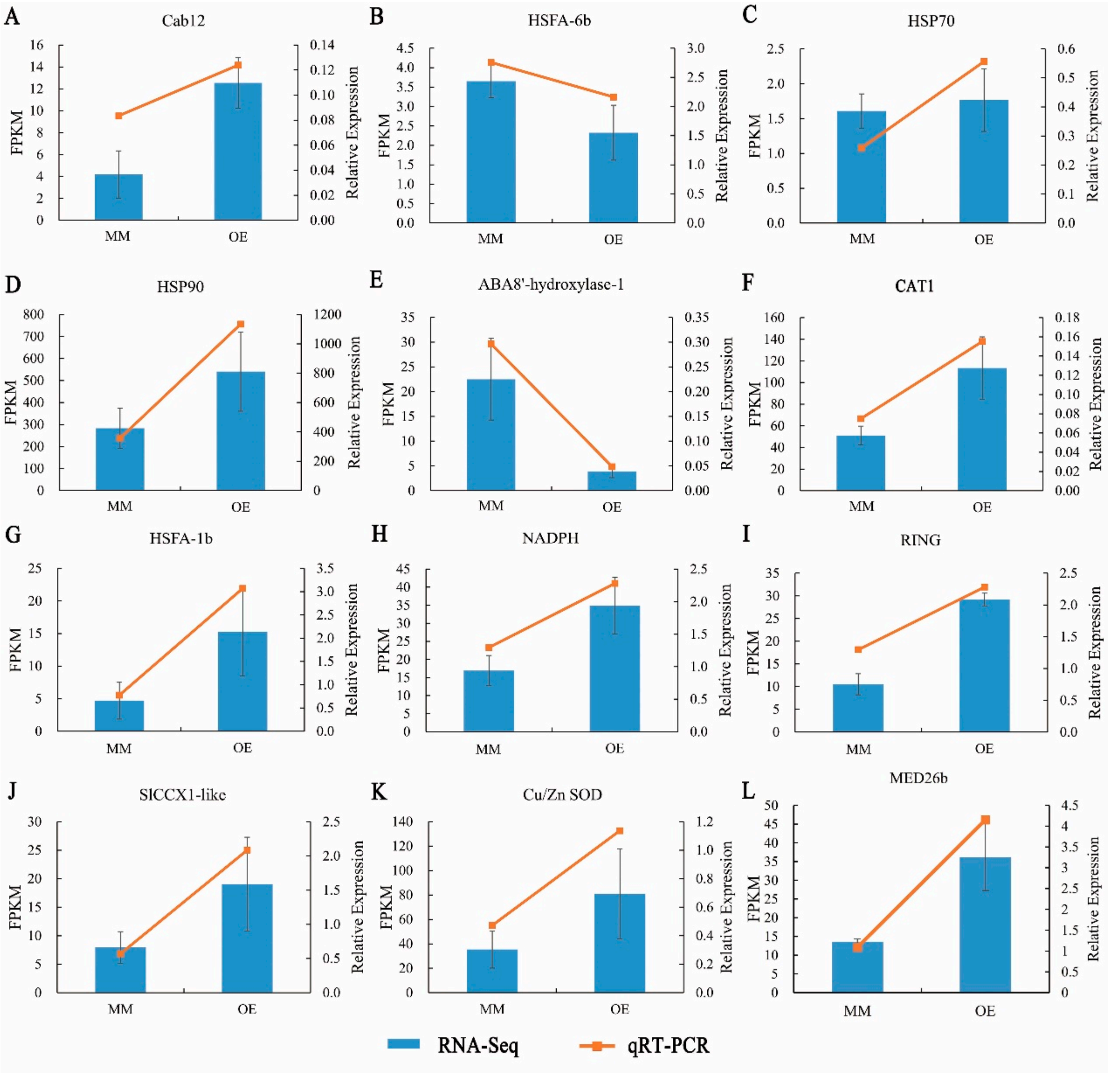

Figure S1 Verifification of RNA-seq results by qRT-PCR (MM-12h vs OE-12h).

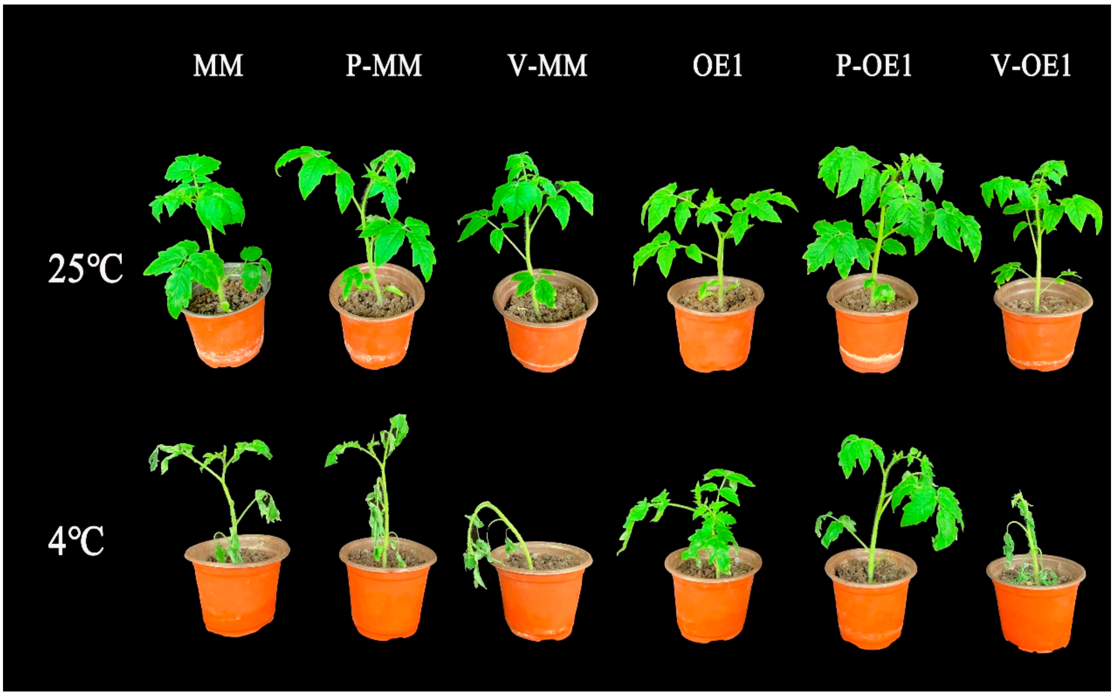

Figure S2 Phenotypic changes of MM, *SlCCX1-like*-silenced MM, OE1, *SlCCX1-like*-silenced OE1 seedlings under chilling stress. MM, control plants; P-MM, empty vector control plants; V-MM, *SlCCX1-like*-silenced MM plants; OE1, overexpression of *SlHSP17.7* plants; P-OE1, empty vector OE1 plants; V-OE1, *SlCCX1-like*-silenced OE1 plants.

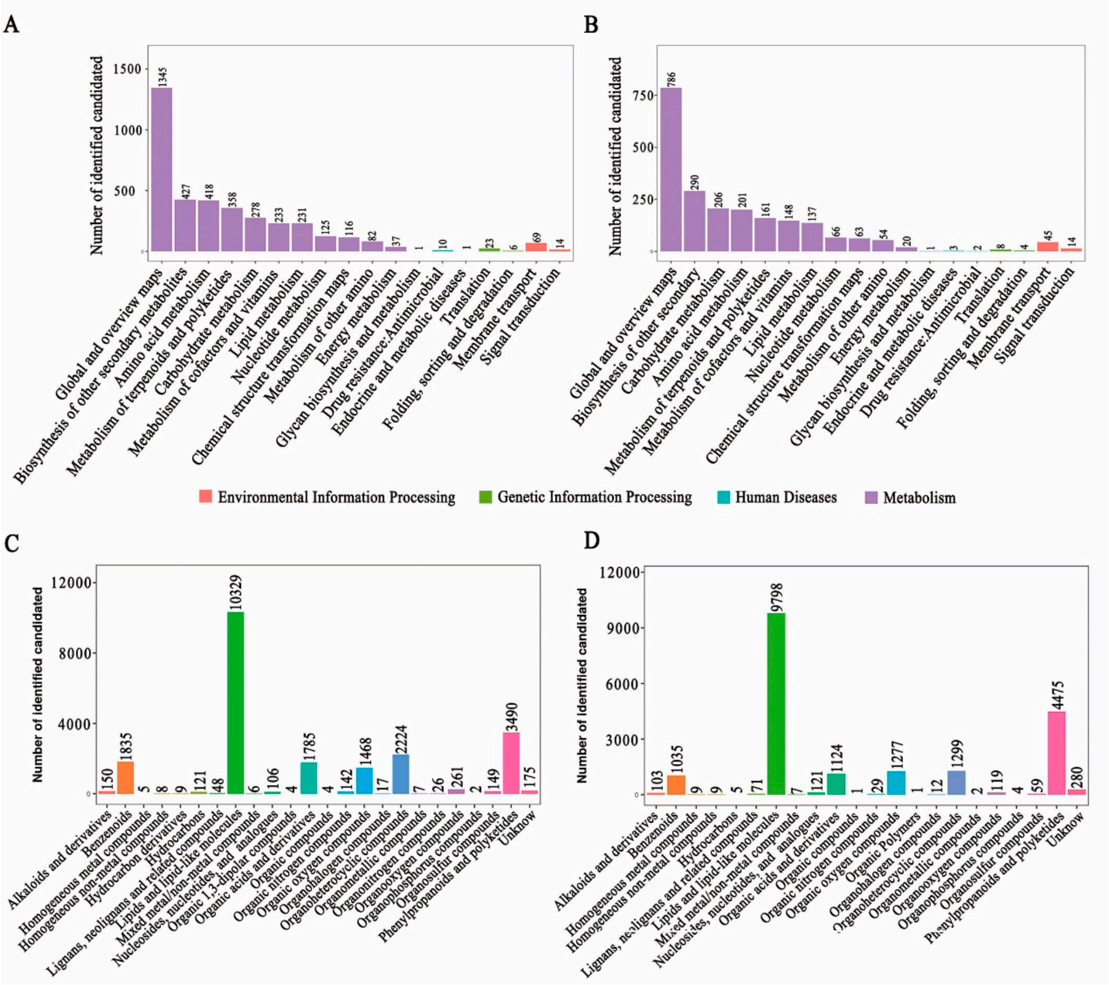

Figure S3 Metabolite identification. A. MS1-KEGG/pos; B. MS1-KEGG/neg; C. MS1-HMDB/pos; D. MS1-HMDB/neg.

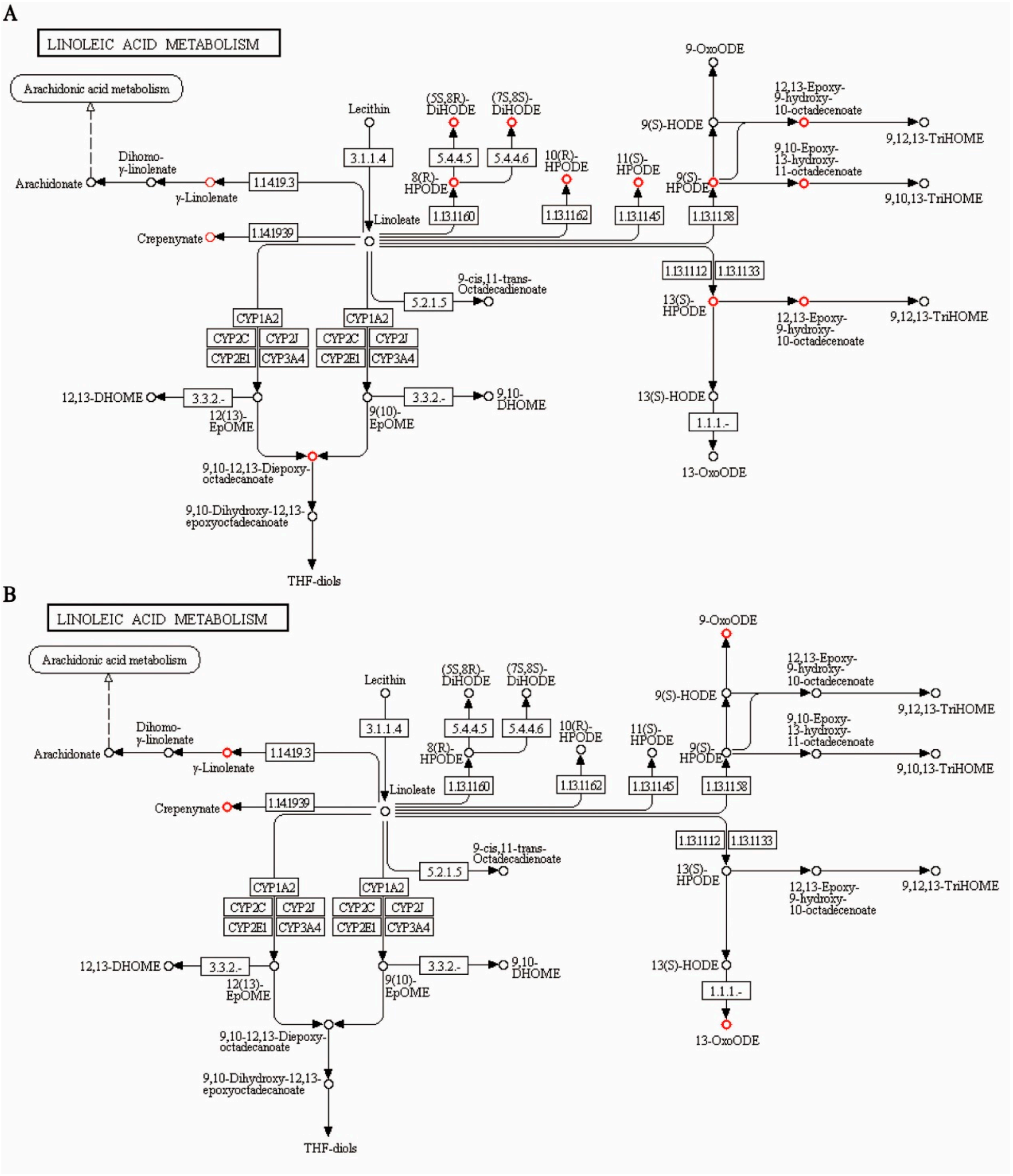

Figure S4 The differential metabolites in linoleic acid metabolism before (A) and after chilling stress for 12 h (B). The red circle indicates the up-regulated expression of this metabolite in OE1 plants.

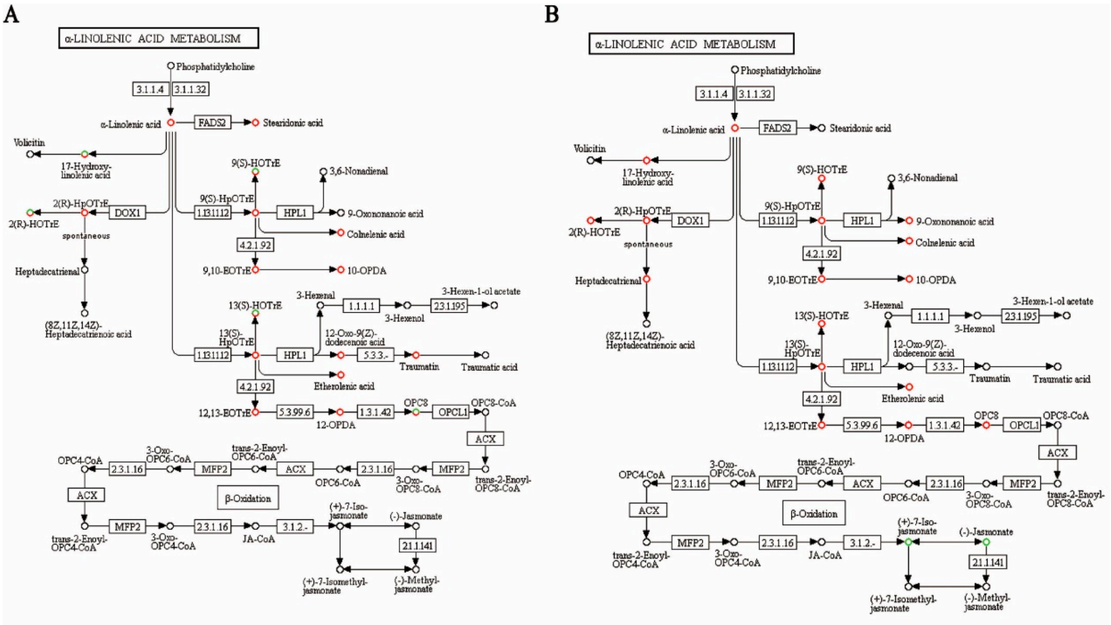

Figure S5 The differential metabolites in alpha−linolenic acid metabolism before (A) and after chilling stress for 12 h (B). The red circle indicates the up-regulated expression of this metabolite in OE1 plants.

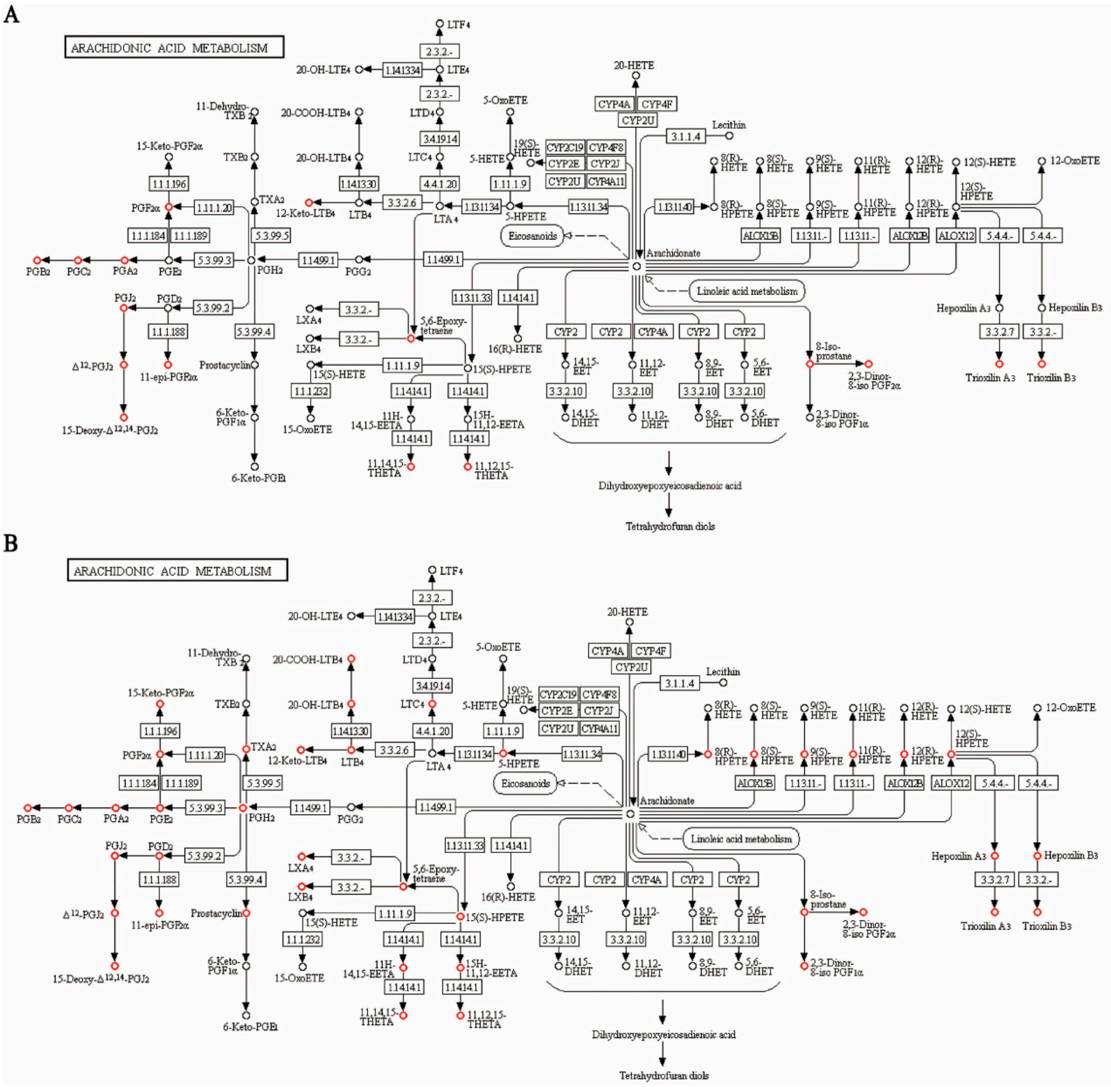

Figure S6 The differential metabolites in arachidonic acid metabolism before (A) and after chilling stress for 12h (B). The red circle indicates the up-regulated expression of this metabolite in OE1 plants.

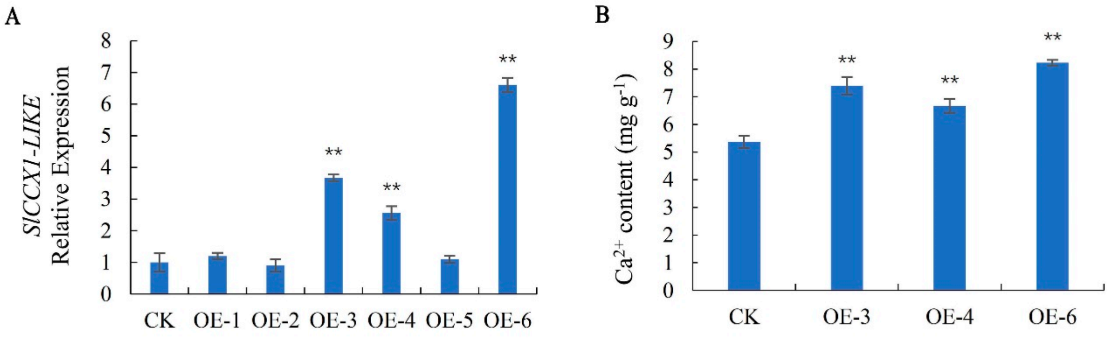

Figure S7 Overexpression of *SlCCX1-like* in Micro-Tom (CK) increased calcium concentration of the leaves. (A) The expression of *SlCCX1-like* in *SlCCX1-like* OE lines. (B) The content of Ca^{2+} in *SlCCX1-like* OE plants.
