# Supplementary material for: SlHSP17.7 Ameliorates Chilling Stress-Induced Damage by Regulating Phosphatidylglycerol Metabolism and Calcium Signal in Tomato Plants
Source: Plants (Basel). 2022 Jul 18;11(14):1865. doi: 10.3390/plants11141865 (PMC9324031; doi:10.3390/plants11141865)
Supplement: Supplementary file 1 [file plants-11-01865-s001.zip › Table S--HSP17.7.pdf]

Table S1: List of primers used in this paper

GeneID	Name	Sequence
Solyc11g005330.1	Actin-F	TGTCCCTATTTACGAGGGTTATGC
Solyc11g005330.1	Actin-R	AGTTAAATCACGACCAGCAAGAT
Solyc06g076540.1	17.7-F	CACCGAAGGAGGAAGGAAAGTGG
Solyc06g076540.1	17.7-R	TTTGCGTTCTCTGGAAGTC
Solyc11g020040.1	HSP70-F	TCAAACTTGAGTGTCCTGCTAT
Solyc11g020040.1	HSP70-R	CAGTGGGCTCATTGATTATTCG
Solyc06g036290.2	HSP90-F	GTGTGGATGTTGATGATATGCC
Solyc06g036290.2	HSP90-R	GACTCGGAAGAACAAAACAACA
Solyc06g053960.2	HSFA-6b-F	AGGAGTGTCTTCATGAGAATGG
Solyc06g053960.2	HSFA-6b-R	CGTCGTTGTTACTTGGATCATC
Solyc06g069730.2	Cab12-F	GGCTTAACCTCTCCTACTTACC
Solyc06g069730.2	Cab12-R	GTCTGATTTTCCAGCATCGTAC
Solyc08g005610.2	ABA8'-hydroxylase-1-F	CTATCAGAAACATCATCCCCGA
Solyc08g005610.2	ABA8'-hydroxylase-1-R	AGTGTACCGGGGAGATTAATTG
Solyc02g079180.1	HSFA-1b-F	GGAACGACAGGCTATAGATGAA
Solyc02g079180.1	HSFA-1b-R	CTCTAATACGTCTAGTTGCCGT
Solyc06g071060.1	NADPHF	TGGAAGCAATGGTGAAGATACT
Solyc06g071060.1	NADPHR	CATTGACACGAATGATCTGTCC
Solyc12g094620.1	CAT1F	AGAAGATACCTGAACGTGTTGT
Solyc12g094620.1	CAT1R	GTTACCCTCTCTGGTGTAGAAC
Solyc02g078460.2	RING-F	CAGGGGTTGAAGCTAAGTTAGA
Solyc02g078460.2	RING-R	TAAAACTGGAACTGTACAGGCA
Solyc11g066390.2	SOD-F	ATGGTCCAACTACGGTTAATGT

Solyc11g066390.2	SOD-R	AGGAGCTCCATGTGTCAATTTA
Solyc07g006370.1	CCX1LIKE-F	CCCGGGTAGGGATGTCTGTTTCTT
Solyc07g006370.1	CCX1LIKE-R	GGCGCGCCAAGTCACCCATAGAGTTT
Solyc10g080930.2	MED26b-F	TGCTGCTGTTGATTGTCC
Solyc10g080930.2	MED26b-R	CCACCAAACTCATCCTTACTC
Solyc10g080930.2	MED26b vigs-F	CCGGATCCATGGCGAAGAGTAGTGGGAC
Solyc10g080930.2	MED26b vigs-R	CCCTCGAGATCTATCCTACTGTTAACTTTGCTCTC
	vigs-F	GATTCTGTGAGTAAGGTTACC
	vigs-R	CTTCAGACACGGATCTAC

Table S2: Differentially expressed genes (DEGs) of photosynthesis before chilling stress (q<0.05, |log2|⩾1)

GeneID	Description	log2 (Fold change)	Regulation
Solyc00g500130.1	photosystem II protein D1 [Nicotiana tabacum]	-1.609853888	down
Solyc00g500329.1	photosystem II protein D1 [Nicotiana tabacum]	-1.629386585	down
Solyc00g500071.1	photosystem II 47 kDa protein [Nicotiana tabacum]	-1.733368334	down
Solyc00g500024.1	photosystem II 47 kDa protein [Nicotiana tabacum]	-1.808817279	down
Solyc06g060340.3	photosystem II subunit S [Solanum lycopersicum]	-1.99790134	down
Solyc00g500056.1	photosystem I P700 apoprotein A2 [Solanum bulbocastanum]	-2.040377707	down
Solyc00g500057.1	photosystem I P700 apoprotein A1 [Solanum bulbocastanum]	-2.471799934	down
Solyc00g500200.1	photosystem II protein D1 [Nicotiana tabacum]	-2.700341438	down
Solyc00g500139.1	photosystem I P700 apoprotein A1 [Solanum bulbocastanum]	-3.61821403	down
Solyc00g500138.1	photosystem I P700 apoprotein A2 [Solanum bulbocastanum]	-3.951049902	down
Solyc00g500136.1	photosynthetic reaction centre, L/M, Photosystem antenna protein-like protein, plastid (chloroplast) [Artemisia annua]	-6.830590817	down
Solyc03g005260.3	ATP sulfurylase 1, chloroplastic [Solanum lycopersicum]	-1.016338005	down
Solyc02g092450.3	calcium-transporting ATPase 2, plasma membrane-type-like isoform X2 [Solanum lycopersicum]	-1.148683903	down
Solyc00g500345.1	ATP synthase subunit 1 (mitochondrion) [Solanum lycopersicum]	-1.232840136	down
Solyc12g089010.3	pre-mRNA-splicing factor ATP-dependent RNA helicase DEAH7-like isoform X2 [Solanum pennellii]	-1.282770093	down
Solyc00g500203.1	ATP synthase CF1 alpha subunit [Solanum bulbocastanum]	-1.349312635	down
Solyc01g104930.3	vesicle-fusing ATPase [Solanum lycopersicum]	-1.383818851	down
Solyc00g500063.1	ATP synthase CF1 beta subunit (chloroplast) [Solanum lycopersicum]	-1.586133228	down
Solyc02g070990.1	chlorophyll a-b binding protein 1, chloroplastic [Solanum lycopersicum]	-1.020989005	down
Solyc12g006140.2	chlorophyll a-b binding protein 5, chloroplastic [Solanum lycopersicum]	-1.090373582	down
Solyc08g066360.3	NADP-dependent malic enzyme isoform X1 [Solanum lycopersicum]	-1.428353872	down

Solyc07g055900.1	ATP-dependent DNA helicase PIF1 [Solanum lycopersicum]	1.044063798	up
Solyc06g071620.3	LOW QUALITY PROTEIN: DExH-box ATP-dependent RNA helicase DExH12-like [Solanum lycopersicum]	1.144314058	up
Solyc06g063330.3	vacuolar H+-ATPase A2 subunit isoform [Solanum lycopersicum]	7.277112144	up
Solyc02g071030.2	chlorophyll a-b binding protein 1B, chloroplastic [Solanum lycopersicum]	1.238903221	up

Table S3: Differentially expressed genes (DEGs) of photosynthesis after chilling stress for 12h (q<0.05, |log2|≥1)

GeneID	Description	log2 (Fold change)	Regulation
Solyc05g010240.4	ruBisCO large subunit-binding protein subunit beta, chloroplastic isoform X2 [Solanum lycopersicum]	-1.171733135	down
Solyc10g006530.4	photosynthetic NDH subunit of lumenal location 3, chloroplastic [Solanum lycopersicum]	-1.164372788	down
Solyc05g007780.3	photosynthetic NDH subunit of lumenal location 2, chloroplastic isoform X1 [Solanum lycopersicum]	-1.170458934	down
Solyc03g062720.3	photosynthetic NDH subunit of subcomplex B 2, chloroplastic [Solanum lycopersicum]	-1.685692476	down
Solyc00g500136.1	photosynthetic reaction centre, L/M, Photosystem antenna protein-like protein, plastid (chloroplast) [Artemisia annua]	-1.824836258	down
Solyc00g500206.1	photosynthetic reaction centre, L/M, Photosystem antenna protein-like protein, plastid (chloroplast) [Artemisia annua]	-2.567216651	down
Solyc00g500054.1	photosynthetic reaction centre, L/M, Photosystem antenna protein-like protein, plastid (chloroplast) [Artemisia annua]	#NUM!	down
Solyc09g076030.4	photosystem II D1 precursor processing protein PSB27-H2, chloroplastic [Solanum lycopersicum]	-1.037646477	down
Solyc12g009200.2	photosystem I chlorophyll a/b-binding protein 6, chloroplastic [Solanum lycopersicum]	-1.224356005	down
Solyc00g500130.1	photosystem II protein D1 [Nicotiana tabacum]	-1.229438205	down
Solyc00g500296.1	photosystem II protein D1 [Nicotiana tabacum]	-1.369999785	down
Solyc09g064500.3	photosystem II reaction center Psb28 protein [Solanum lycopersicum]	-1.442698311	down
Solyc00g500024.1	photosystem II 47 kDa protein [Nicotiana tabacum]	-1.765651186	down
Solyc00g500136.1	photosynthetic reaction centre, L/M, Photosystem antenna protein-like protein, plastid (chloroplast) [Artemisia annua]	-1.824836258	down
Solyc00g500208.1	photosystem I P700 apoprotein A2 [Solanum bulbocastanum]	-1.860966071	down
Solyc00g500071.1	photosystem II 47 kDa protein [Nicotiana tabacum]	-1.864580717	down
Solyc06g060340.3	photosystem II subunit S [Solanum lycopersicum]	-2.009254498	down
Solyc00g500329.1	photosystem II protein D1 [Nicotiana tabacum]	-2.107967508	down
Solyc00g500056.1	photosystem I P700 apoprotein A2 [Solanum bulbocastanum]	-2.29436619	down

Solyc00g500209.1	photosystem I P700 apoprotein A1 [Solanum bulbocastanum]	-2.299832152	down
Solyc00g500206.1	photosynthetic reaction centre, L/M, Photosystem antenna protein-like protein, plastid (chloroplast) [Artemisia annua]	-2.567216651	down
Solyc00g500057.1	photosystem I P700 apoprotein A1 [Solanum bulbocastanum]	-2.793768426	down
Solyc00g500054.1	photosynthetic reaction centre, L/M, Photosystem antenna protein-like protein, plastid (chloroplast) [Artemisia annua]	#NUM!	down
Solyc04g040160.4	protochlorophyllide-dependent translocon component 52, chloroplastic [Solanum lycopersicum]	-1.069442561	down
Solyc12g009200.2	photosystem I chlorophyll a/b-binding protein 6, chloroplastic [Solanum lycopersicum]	-1.224356005	down
Solyc06g053980.3	chlorophyllase-2, chloroplastic [Solanum lycopersicum]	-1.295304466	down
Solyc09g076030.4	photosystem II D1 precursor processing protein PSB27-H2, chloroplastic [Solanum lycopersicum]	-1.037646477	down
Solyc04g064670.3	psbP domain-containing protein 4, chloroplastic [Solanum lycopersicum]	-1.062748351	down
Solyc04g009420.3	psbP domain-containing protein 2, chloroplastic [Solanum lycopersicum]	-1.339528305	down
Solyc09g064500.3	photosystem II reaction center Psb28 protein [Solanum lycopersicum]	-1.442698311	down
Solyc05g055700.3	2-alkenal reductase (NADP(+)-dependent) [Solanum lycopersicum]	-1.120839689	down
Solyc07g005390.3	NADP-dependent glyceraldehyde-3-phosphate dehydrogenase [Solanum lycopersicum]	-1.201293201	down
Solyc10g007600.3	glycolate oxidase [Solanum lycopersicum]	-1.091505684	down
Solyc06g005950.3	ATP-dependent zinc metalloprotease FTSH 9, chloroplastic [Solanum lycopersicum]	-1.047236831	down
Solyc12g042950.2	plastidic ATP/ADP-transporter [Solanum lycopersicum]	-1.077806677	down
Solyc00g500332.1	ATP synthase CF1 alpha subunit [Solanum bulbocastanum]	-1.134744834	down
Solyc07g055320.4	ATP-dependent zinc metalloprotease FTSH 2, chloroplastic [Solanum lycopersicum]	-1.161824464	down
Solyc02g062780.4	ATP-dependent DNA helicase DDM1-like isoform X1 [Solanum lycopersicum]	-1.172198706	down
Solyc12g010870.3	ATP synthase I-like protein [Solanum lycopersicum]	-1.248645187	down
Solyc02g094100.4	ATPase family AAA domain-containing protein FIGL1 [Solanum lycopersicum]	-2.316528145	down
Solyc09g065620.3	chlorophyllase-2, chloroplastic isoform X1 [Solanum lycopersicum]	1.493444712	up
Solyc06g069730.3	chlorophyll a-b binding protein Cab12 [Solanum lycopersicum]	1.581458337	up
Solyc06g071060.1	NADPH-dependent aldehyde reductase-like protein, chloroplastic [Solanum lycopersicum]	1.044281288	up

Solyc12g010960.2	2-alkenal reductase (NADP(+)-dependent)-like [Solanum lycopersicum]	2.172239946	up
Solyc09g082870.3	putative calcium-transporting ATPase 13, plasma membrane-type [Solanum lycopersicum]	1.203052028	up
Solyc02g084900.2	AAA-ATPase At2g18193-like [Solanum lycopersicum]	1.265429595	up
Solyc02g092450.3	calcium-transporting ATPase 2, plasma membrane-type-like isoform X2 [Solanum lycopersicum]	1.68166333	up
Solyc03g033840.4	AAA-ATPase At3g50940-like [Solanum lycopersicum]	1.78374191	up
Solyc06g071620.3	LOW QUALITY PROTEIN: DExH-box ATP-dependent RNA helicase DExH12-like [Solanum lycopersicum]	1.864074807	up
Solyc12g017700.2	pre-mRNA-splicing factor ATP-dependent RNA helicase DEAH1 isoform X2 [Solanum lycopersicum]	2.185965998	up
Solyc00g500140.1	ATP synthase CF1 epsilon subunit [Solanum bulbocastanum]	2.458253873	up
Solyc12g089010.3	pre-mRNA-splicing factor ATP-dependent RNA helicase DEAH7-like isoform X2 [Solanum pennellii]	3.125920245	up
Solyc06g063330.3	vacuolar H+-ATPase A2 subunit isoform [Solanum lycopersicum]	6.18292852	up

Table S4 List of co-expressed MED and HSP genes.

Gene	Description	Rank
**HSP17.7**	17.7 kDa class II heat shock protein [Source:UniProtKB/Swiss-Prot;Acc: O81822]	N/A
HSP17.6C	17.6 kDa class I heat shock protein 3 [Source:UniProtKB/Swiss-Prot;Acc: P13853]	1
HSP17.6	17.6 kDa class II heat shock protein [Source:UniProtKB/Swiss-Prot;Acc: P29830]	2
HSP17.4A	17.4 kDa class I heat shock protein [Source:UniProtKB/Swiss-Prot;Acc: P19036]	3
MED37C	Probable mediator of RNA polymerase II transcription subunit 37c [Source:UniProtKB/Swiss-Prot;Acc:Q9LHA8]	4
HSP23.6	HSP23.6-MITO [Source:UniProtKB/TrEMBL;Acc:A0A178USN5]	5
HSP17.4B	17.4 kDa class III heat shock protein [Source:UniProtKB/Swiss-Prot;Acc: Q9SYG1]	6
HSP22.0	22.0 kDa heat shock protein [Source:UniProtKB/Swiss-Prot;Acc:Q38806]	7
HSP17.6B	17.6 kDa class I heat shock protein 2 [Source:UniProtKB/Swiss-Prot;Acc: Q9ZW31]	8
HSP23.5	23.5 kDa heat shock protein, mitochondrial [Source:UniProtKB/Swiss-Prot;Acc:Q9FGM9]	9
HSP70-3	Heat shock protein 70 (Hsp 70) family protein [Source:UniProtKB/TrEMBL;Acc:A0A178VI76]	10
HSP15.7	15.7 kDa heat shock protein, peroxisomal [Source:UniProtKB/Swiss-Prot;Acc:Q9FHQ3]	11
HSP21	HSP21 [Source:UniProtKB/TrEMBL;Acc:A0A178UVU5]	12
MED37D	Probable mediator of RNA polymerase II transcription subunit 37c	13

	[Source:UniProtKB/Swiss-Prot;Acc:P22954]	
HSP26.5	26.5 kDa heat shock protein, mitochondrial [Source:UniProtKB/Swiss-Prot;Acc:Q9SSQ8]	14
MED37E	Probable mediator of RNA polymerase II transcription subunit 37e [Source:UniProtKB/Swiss-Prot;Acc:P22953]	15
HSP18.1	18.1 kDa class I heat shock protein [Source:UniProtKB/Swiss-Prot;Acc:P19037]	16
CLPB1	Chaperone protein ClpB1 [Source:UniProtKB/Swiss-Prot;Acc:P42730]	17
AT1G54400	HSP20-like chaperones superfamily protein [Source:UniProtKB/TrEMBL;Acc:F4HWW7]	18
AT5G20970	HSP20-like chaperones superfamily protein [Source:UniProtKB/TrEMBL;Acc:F4K6X6]	19
HSP70-5	Hsp70b [Source:UniProtKB/TrEMBL;Acc:A0A178W9N7]	20
